# Mucopolysaccharidosis-Plus Syndrome, a Rapidly Progressive Disease: Favorable Impact of a Very Prolonged Steroid Treatment on the Clinical Course in a Child

**DOI:** 10.3390/genes13030442

**Published:** 2022-02-28

**Authors:** Martha Caterina Faraguna, Francesca Musto, Viola Crescitelli, Maria Iascone, Luigina Spaccini, Davide Tonduti, Tiziana Fedeli, Gaia Kullmann, Francesco Canonico, Alessandro Cattoni, Fabiola Dell’Acqua, Carmelo Rizzari, Serena Gasperini

**Affiliations:** 1 Università degli Studi Milano-Bicocca, 20100 Milan, Italy; m.faraguna@campus.unimib.it (M.C.F.); f.musto@campus.unimib.it (F.M.); 2 Department of Pediatrics, Università degli Studi Milano-Bicocca, Fondazione MBBM, San Gerardo Hospital, Via Pergolesi 33, 20900 Monza, Italy; vcrescitelli@fondazionembbm.it (V.C.); a.cattoni1@campus.unimib.it (A.C.); fdellacqua@fondazionembbm.it (F.D.); carmelo.rizzari@gmail.com (C.R.); 3 Laboratory of Genetic Medicine, ASST Papa Giovanni XXIII, 24127 Bergamo, Italy; maria.iascone@gmail.com; 4 Clinical Genetics Unit, Department of Obstetrics and Gynecology, V. Buzzi Children’s Hospital, University of Milan, 20100 Milan, Italy; luigina.spaccini@asst-fbf-sacco.it; 5 Pediatric Neurology Unit, V. Buzzi Children’s Hospital, 20100 Milan, Italy; davide.tonduti@asst-fbf-sacco.it; 6 C.O.A.L.A (Center for Diagnosis and Treatment of Leukodystrophies), V. Buzzi Children’s Hospital, 20100 Milan, Italy; 7 Neonatal Intensive Care Unit, FMBBM, San Gerardo Hospital, 20900 Monza, Italy; fedelitiziana@gmail.com; 8 Child Neuropsychiatrist Unit, San Gerardo Hospital, 20900 Monza, Italy; g.kullman@asst-monza.it; 9 Department of Neuroradiology, Università degli Studi Milano Bicocca, San Gerardo Hospital, 20900 Monza, Italy; f.canonico@asst-monza.it

**Keywords:** mucopolysaccharidosis-plus, lysosomal storage disease, secondary hemophagocytic lymphohistiocytosis, leukoencephalopathy

## Abstract

Mucopolysaccharidosis-plus syndrome (MPS-PS) is a novel autosomal recessive disorder caused by a mutation in the *VPS33A* gene. This syndrome presents with typical symptoms of mucopolysaccharidosis, as well as congenital heart defects, renal, and hematopoietic system disorders. To date, twenty-four patients have been described. There is no specific therapy for MPS-PS; clinical management is therefore limited to symptoms management. The clinical course is rapidly progressive, and most patients die before 1–2 years of age. We describe a currently 6-year-old male patient with MPS-PS presenting with multiorgan involvement. Symptoms started at four months of age when he progressively suffered from numerous acute and potentially life-threatening events. When he was two years old, he developed secondary hemophagocytic lymphohistiocytosis (HLH), which was successfully treated with steroids. To date, this child represents the oldest patient affected by MPS-PS described in the literature and the first one presenting with a life-threatening secondary HLH. The prolonged steroid treatment allowed a stabilization of his general and hematological conditions and probably determined an improvement of his psychomotor milestones and new neurological acquisitions with an improvement of quality of life. HLH should be suspected and adequately treated in MPS-PS patients presenting with suggestive symptoms of the disease. The usefulness of a prolonged steroid treatment to improve the clinical course of children with MPS-PS deserves further investigation.

## 1. Introduction

### 1.1. Background

Lysosomal storage diseases (LSD) are a group of inherited metabolic disorders characterized by a lysosomal enzyme deficiency, with subsequent accumulation of substrate [1]. More than fifty LSD have been described, and their overall prevalence is around 1:5000–1:7000 [2,3]. These disorders are progressive, although the introduction of new therapies has positively affected the natural history of some diseases.

Mucopolysaccharidosis (MPS) is a rare LSD caused by the deficiency of lysosomal enzymes involved in the degradation of glycosaminoglycans (GAGs), such as dermatan sulfate, heparan sulfate, keratan sulfate, chondroitin sulfate, and hyaluronic acid. The storage of GAGs in different organs is progressive and leads to a broad spectrum of clinical manifestations. There are seven different MPS syndromes caused by deficiency of one of eleven possible enzymes [1]. Clinical presentation, age at onset, clinical course, diagnosis, and treatment vary from one type of MPS to another, and the spectrum of clinical manifestation varies within the same type of MPS. Therefore, a multidisciplinary team is necessary for management and treatment. Diagnosis is based on clinical manifestations, urine GAGs analysis, and enzymatic determination. Their identification is essential to initiate early treatment [4]. Current therapy consists of Enzyme Replacement Therapy (ERT) for MPS I, II, IVA, VI, and VII. As ERT does not cross the blood–brain barrier, hematopoietic stem cell transplantation (HSCT) has become the standard of care in MPS I-Hurler— the most severe form of MPSI with Central Nervous System (CNS) involvement—whenever patients are aged two or less and show a demonstrated mild (IQ > 70) or absent cognitive impairment [5]. These therapies are not curative but can slow down the progression of the disease. Accordingly, more effective and feasible therapies are required. Clinical trials for the efficacy of gene therapy in MPS I are ongoing [6].

### 1.2. MPS-PS

Mucopolysaccharidosis-plus syndrome (MPS-PS) is an autosomal recessive disorder recently described within LSD. The only mutation reported so far is the c.1492C>T (p.R498W) missense mutation in the *VPS33A* gene [7,8,9,10].

Twenty-four patients have been described to date: twenty-two patients among the Yakut population [7,9] and two from Turkey [8]. All patients from Yakutia (northeast Siberia, the Sakha Republic in the Russian Federation) were born to nonconsanguineous healthy parents. The incidence rate of MPS-PS among Yakuts is predicted as 1 per 12–15,000 births [7], while allele frequency has been found to be 1 in 110 in a random healthy population [8]. In fact, the Yakut population is characterized by a high degree of genetic homogeneity due to geographical isolation and low migration. These factors determined the founder effect and a high frequency of hereditary diseases [7,11,12].

The two Turkish patients described by Dursun et al. are two female siblings born to first-cousin parents. The findings of this rare variant in Yakut and Turkish patients are consistent with the Turkic ancestry of the present Yakut population [13,14].

Patients affected by MPS-PS share most signs and symptoms of MPS: coarse facial features (short neck and nose, prominent forehead, epicanthal folds, thick hair, excessive hair growth, periorbital puffiness, full lips, macroglossia), thick skin, skeletal abnormalities (barrel-shaped chest, pectus carinatum, kyphosis, lordosis, multiple dysostosis, joints contracture, stiffness of the elbow, wrist, hip, knee and ankle joints, finger phalangeal swelling, limitation of motion of fingers, claw-hand deformities, deep palmar furrows), abdominal hernia, respiratory system abnormalities (bronchial obstruction, noisy breathing, shortness of breath, frequent respiratory tract infections, snoring, sleep apneas), cardiovascular pathologies (valve insufficiency, congenital heart defects, pulmonary hypertension), and neurologic abnormalities (hydrocephalus, hypotonia, poor tendon reflexes, developmental delay).

Besides similar multiorgan involvement of MPS, the clinical presentation of MPS-PS shows some specificities: rapidly evolving heart defects, renal, and hematopoietic disorders.

Among the MPS-PS patients described [7,8,9,10], kidney involvement consists of progressive proteinuria, nephromegaly, nephritic, and nephrotic syndrome. Kidney biopsy revealed segmental or global sclerosis, periglomerular fibrosis, and atrophy of tubules [10]. These complications were treated with ACE inhibitors [7].

Rapidly evolving heart defects observed in MPS-PS patients include severe valve insufficiency, hypertrophic cardiomyopathy, and congenital heart defects, such as patent ductus arteriosus and atrial septal defect [8]. These abnormalities often require surgical correction.

A significant difference with MPS patients is also hematologic involvement; anemia, thrombocytopenia, leukocytopenia, and coagulopathy with episodic intestinal bleeding are reported. In most patients who underwent bone marrow biopsy, hypoplastic bone marrow was found. Pavlova et al. report increased serum levels of IgM and decreased IgG [9], as well as neutropenia, which accounts for impaired humoral immunity and susceptibility to recurrent respiratory infections [7,8,9].

Respiratory involvement is often severe, with Computerized Tomography (CT) scans showing consolidations, ground-glass opacities, peribronchial thickenings, and hyperinflation of parenchyma. In bronchoalveolar lavage specimens, hemosiderin and lipid-loaded macrophages were detected. At postmortem examination, fibrosis and iron-full macrophages were found [10].

There is no definite neurological phenotype, albeit Central Nervous System (CNS) is always involved. Psychomotor delay, hypotonia, poor tendon reflexes, and nystagmus are reported, but no neuroimaging nor neurophysiological data have been described so far except for one patient for whom brain Magnetic Resonance Imaging (MRI) and CT were performed to disclose delayed myelination and basal ganglia calcification [8]. One case of cerebral calcification in the white matter and one case of normal cytoarchitecture and perivascular and focal parenchymal edema at postmortem examination are reported [8,10].

There is no specific therapy for MPS-PS. Clinical management is limited to supportive and symptomatic treatment, such as antibiotics for infections, ACE-inhibitors for proteinuria, and blood transfusions for anemia.

The prognosis of MPS-PS is generally unfavorable, and most patients die at 10–20 months of age [1]. The main cause of death is respiratory insufficiency due to infections.

No birth defects have been identified by ultrasound investigation during pregnancy [7]. Definitive diagnosis is obtained by genetic testing.

### 1.3. Pathogenesis and Genetic Analysis

As previously said, MPS-PS is caused by the missense mutation p.R498W (the only one described so far in all patients) in the *VPS33A* gene and is inherited by an autosomal recessive pattern. All patients described had heterozygous carrier parents [7,8,9,10].

P.R498W mutation in the *VPS33A* gene affects GAGs metabolism; however, substrate accumulation is not caused by decreased lysosomal enzyme activity [7,8,9,10]. This is the main biochemical difference with MPS.

VPS33A is a core component of the HOPS (Homotypic Fusion and Protein Sorting) and the CORVET (Class C core Vacuole/Endosome Tethering) complexes which have essential functions in the endocytic trafficking pathway and for vacuolar fusion. Vacuolar protein sorting (VPS) mutants were first isolated in studies on yeast with a large collection of complementation groups containing more than 40 genes [15]. Mutations of the CORVET and HOPS subunits were subdivided into several classes: mutations of the core subunits VPS11, VPS16, VPS18, and VPS33 showed the most severe phenotype (lack of normal vacuoles), mutations of HOPS subunits (VPS39 and VPS41) showed fragmented vacuoles, and mutations of CORVET subunits (VPS8 and VPS3) showed vacuoles similar to wild type [16]. These studies showed that mutations in the core subunits (VPS11, VPS16, VPS18, and VPS33) eliminate both CORVET and HOPS complexes and therefore have the most severe effects on vacuolar biogenesis. Isolated mutations in CORVET subunits or HOPS taken individually seem to have very little effect on vacuole morphology. Unlike yeast, in humans, the alteration in the core component leads to a severe pathogenetic mechanism which can be lethal [15].

Patients with MPS-PS with a mutation in the *VPS33A* gene, in fact, show numerous features not shared by other forms of MPS and probably reflect the central role of the HOPS complex in cell physiology [9]. As Pavlova et al. have demonstrated, cultured fibroblast from Yakut patients with MPS-PS has morphologic changes: vacuolation with disordered endosomal/lysosomal compartments and, common to sphingolipid diseases, abnormal endocytic trafficking of lactosylceramide. Endocytic and autophagic pathways are normal [7,8]. In one patient, urine glycosaminoglycan analysis revealed a pathologic excess of sialylated conjugated, despite the normal activity of neuraminidase [9]. Extremely high levels of plasma heparan sulfate are reported [8]; however, the activities of lysosomal acid hydrolases implicated in MPS diseases are commonly within the healthy reference ranges in both blood and fibroblasts of patients with MPS-PS [9]. Kondo et al. reported higher acidity of lysosomes in patient-derived fibroblasts and *VPS33A*-depleted cells, suggesting that lysosomal overacidification causes lysosomal dysfunction and storage of undegraded substrates [8]. Detailed pathophysiology remains to be cleared.

Therefore MPS-PS should be considered in a patient with MPS features but normal lysosomal enzyme activity. Plasma and urine GAGs may be increased or normal [9,10].

Pavlova et al. suggested two possible therapeutical targets. Firstly, considering that several unrelated sphingolipid storage diseases have a common sphingolipid trafficking defect, fibroblasts were exposed to proteasome inhibitor bortezomib for correction of the trafficking. Secondly, similar results were obtained by exposing fibroblasts to the glucosylceramide synthesis inhibitor eliglustat, a chaperone drug approved for the treatment of Gaucher type 1 disease [9].

## 2. Case Report

We present the first case of a young boy affected by MPS-PS who developed a secondary hemophagocytic lymphohistiocytosis (HLH) during septic shock due to pneumonia.

This thirteen-month-old boy came to our attention for the diagnostic workup of neurodevelopmental delay. He is the second child of consanguineous healthy parents (first cousins) of Moroccan origin. Two previous miscarriages are reported. Pregnancy and first months of life were referred to as unremarkable. At four months of age, the patient developed eye nystagmus and growth stunt. At six months of age, nystagmus was no longer evident, but neurological examination showed mild neurodevelopmental delay, global hypotonia, and hyporeflexia. The child underwent a cerebral MRI revealing myelination delay and thin corpus callosum. Electroneuronography, performed at 13 months of age, highlighted a defect in nerve conduction consistent with a demyelinating sensorimotor neuropathy; peripheral and retrocochlear hearing impairment was also documented at brainstem auditory evoked potentials. Further findings were tubulopathy with low molecular weight proteinuria, reduced electrolytes excreted fractions, cardiac wall thickness, non-autoimmune subclinical hypothyroidism, and normocytic anemia.

Over time the patient developed coarse facial features (short nose and neck, prominent forehead, epicanthal folds, telecanthus, macroglossia, full lips, pronounced philtrum), scaphocephaly, pectus carinatum, lumbar kyphosis, and umbilical hernia.

In addition, the patient showed a neurocognitive picture consistent with developmental delay; he acquired a sitting position at 10 months of age, babbling at 11 months, he was able to walk with aid at 22 months. His fine motor skills were limited because of joint stiffness and claw fingers and resulted in clumsiness and difficulty in object manipulation. Exertional dyspnea influenced endurance. The moderate cognitive delay was evident.

Overall, the clinical features and multisystemic involvement became strongly suggestive of a lysosomal storage disorder.

Multiple metabolic investigations were performed; in particular, urinary oligosaccharides and mucopolysaccharides were normal, while excretion of conjugated sialic acid was increased (479 mmol/mole of creatinine, normal value < 148), as well as urinary sulfatides.

Molecular analysis for mucopolysaccharidosis IV and enzymatic analysis for mucolipidosis I and II were negative.

With a high suspicion of lysosomal storage disease, at 23 months of age, the whole exome sequencing was performed, and a homozygotic mutation (p.R498W) in the *VPS33A* gene was detected.

The patient progressively developed multiple organ involvement, and his clinical condition worsened because of the progressive storage. He developed a severe mitral insufficiency with atrial dilatation requiring increasing doses of carvedilol, furosemide, captopril, and spironolactone. Because of worsening of electrolyte imbalances caused by medications, he was started on oral sodium chloride and potassium supplementation.

Given a long-standing history of iron-refractory hyporegenerative microcytic anemia (Hb 7.6 g/dl MCV 74 fL, reticulocytes 34.000/cmm) and the finding of transient mild thrombocytopenia (PLT 125.000/cmm), the patient underwent a bone marrow aspiration that showed a hypocellular bone marrow with very few myeloid precursors. This result was not conclusive. Peripheral white blood count was normal. Further investigation included specific tests to exclude red cell membrane defects (such as Eosin-5-maleimide (EMA) binding test, Pink test, and Osmotic Fragility Test).

In addition, recurrent infections of the upper respiratory airway and several potentially life-threatening events occurred: cardiac arrest during sedation for bone marrow aspiration and MRI imaging, septic shock, renal insufficiency secondary to colitis by Clostridium difficile, and multiple episodes of pneumonia. Given the history of recurrent infections, immunologic assays were performed (Immunoglobulins, Dihydrorhodamine, and Lymphocyte subpopulations), which turned up as normal.

At the age of two years, the patient was admitted to our Pediatric Department for pneumonia complicated by respiratory insufficiency requiring orotracheal intubation; ten days later, the patient developed septic shock associated with clinical and laboratory findings suggestive for HLH: fever, high levels of ferritin, triglycerides, two cytopenias (anemia and thrombocytopenia,) and splenomegaly. The patient underwent a bone marrow biopsy that highlighted reduced cellularity with stromal damage and reinforcement of reticulin fibers.

HLH was treated with high doses of intravenous (i.v.) dexamethasone, and the patient required multiple blood transfusions. Genetic testing ruled out primary HLH.

The patient’s clinical conditions dramatically improved once he was started on steroid therapy, while they worsened again when steroid dosage was significantly reduced. Accordingly, a slow tapering of oral dexamethasone (0.2 mg/day every 2–4 weeks) appeared to be a successful strategy (Table 1). As the patient developed severe and persistent hyponatremia over time (nadir, Na 122 mEq/L, range 135-145 mEq/L), he was started on oral supplementation with sodium chloride. After ten months of treatment with dexamethasone, the patient was switched to the equivalent dose of oral hydrocortisone (HC), given its greater mineralocorticoid activity. Once sodium values normalized, further slow decalage in the dose of HC was undertaken until the substitutive dose of 12.5 mg/sq was achieved, thirty months later. After 52 weeks of HC at substitutive dose, in the attempt to discontinue steroid therapy, the patient underwent a low-dose Synacthen test that highlighted a deep and persistent iatrogenic suppression of the hypothalamic-pituitary-adrenal axis (cortisol peak 0.6 mcg/dL). Thus, substitutive HC treatment has been further reduced, and it is still ongoing.

**Figure 1:**
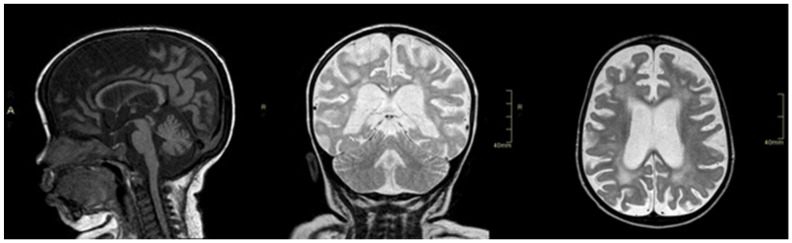
From left to right: sagittal T1 image (cortical-subcortical and corpus callosum atrophy), coronal T2 image (cortical-subcortical atrophy and hyperintensity T2 white matter), axial T2 image (cortical-subcortical atrophy and hyperintensity T2 white matter).

The prolonged hospitalization needed for the management of HLH caused a significant deterioration of neurological skills with loss of postural control. The patient developed progressive dysphagia; therefore, a nasogastric tube was placed. Control MRI revealed global brain atrophy, a severe supratentorial white matter involvement that appeared hyperintense on T2wi and hypointense on T1wi, with relative sparing of U-fibers, pyramidal tracts, and corpus callosum, which was diffusely atrophic but normal intense (Figure 1). After the resolution of the acute phase, neurological skills progressively improved, and at 30 months of age, he was able to walk again with support and to pronounce a few words.

Apparently, with steroid therapy, the patient showed not only a remission of hematologic disease but also a stabilization of the natural history of MPS-PS. Events requiring hospitalization significantly reduced, as well as respiratory support (Continuous Positive Airway Pressure, CPAP) and enteral feeding; in addition, new neurological skills have been acquired. 

**Figure 2:**
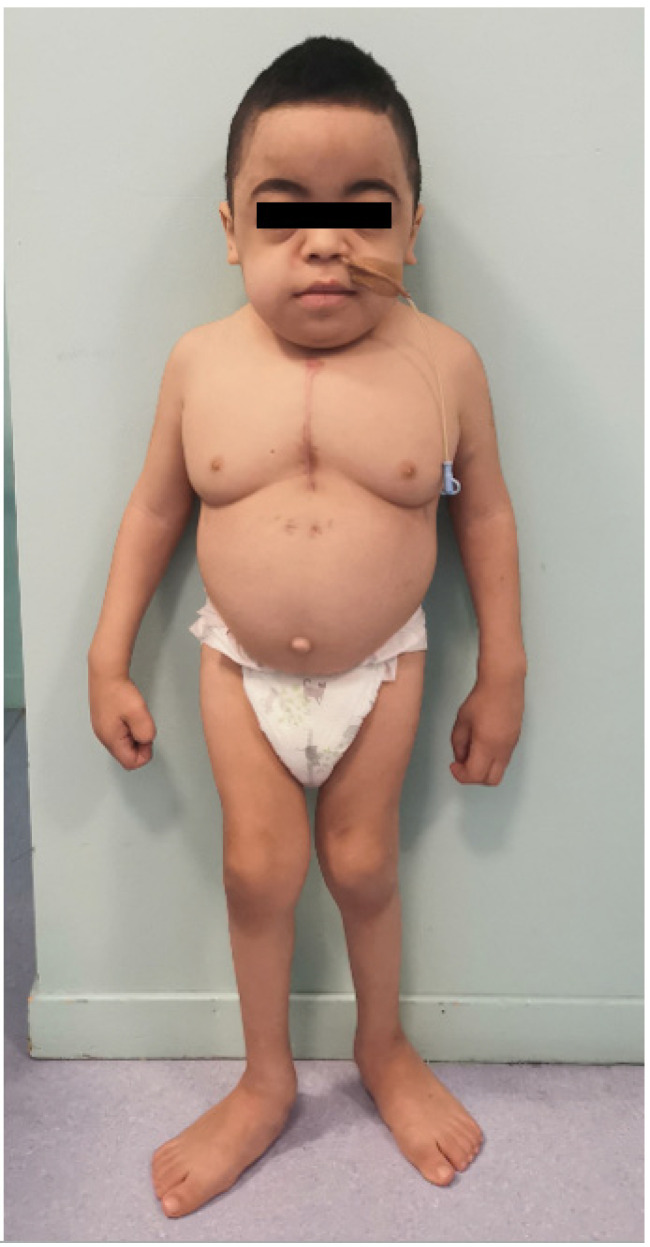
Clinical phenotype of the patient at six years old.

Currently, the patient is six years old. His height and weight are below the third percentile, and his head circumference is between the 75th and 90th percentile. His main comorbidities are glomerular proteinuria, hypothyroidism, and developmental delay (Figure 2). He has recently had a correction of the severe mitral valve insufficiency with the placement of a mechanical valve.

He walks with a wide gait, climbs up and down stairs, pronounces only a few words but has good nonverbal communication. His medications include hydrocortisone, carvedilol, furosemide, ramipril, spironolactone, warfarin, levothyroxine, potassium, vitamin D, and iron supplementation. He requires CPAP during nighttime and is occasionally tube fed.

## 3. Discussion

The clinical history of the first two years of the patient’s life consists of a rapidly progressive multiorgan involvement as described in MPS-PS, unlike patients affected by other lysosomal storage disorders where the symptoms are less aggressive in the first years of life.

We describe the first case of MPS-PS who developed a secondary HLH, which was treated with steroid therapy for a very long period.

The natural history of this patient’s disease is similar to that described for other patients, except for longer survival. Despite the description of neurological involvement in all patients, the literature lacks detailed neuroradiological and neurophysiological data. In our case, we were able to state that the neurological picture was related to a progressive leukoencephalopathy with peripheral demyelinating neuropathy. The clinical neurological picture, with concomitant signs of central and peripheral white matter involvement, and the localization of white matter abnormalities, supratentorial with predominant involvement of periventricular and deep white matter, are typical findings of another LSD, namely Metachromatic Leukodystrophy (MLD) caused by Arylsulfatase A deficiency [17]. MPS-PS is probably caused by the dysfunction of several lysosomal hydroxylases; our observation raises the question if, based on what happens in Multiple Sulfatase Deficiency (MSD) or MLD [18], the neurological picture of MPS-PS could be similar: in fact, urinary sulfatides are elevated in our patient. On the basis of the few studies reported so far, we cannot exclude a pathogenetic role of the other genes involved in the core subunits governing the assembly of both CORVET and HOPS complexes. Further studies in animal models (eukaryotes) to better understand the pathophysiology are needed and could lead to future therapeutical approaches. The correlation between prolonged steroid treatment and good clinical outcomes can lead to a hypothesis of a possible role of inflammation as one of the pathogenic cascade mechanisms involved in LSD [19,20,21]. In LSD, there is a storage of substrate, which triggers an inflammatory response that is not self-limiting, and once triggered, it creates a vicious cycle involving oxidative stress with the production of Reactive Oxygene Species (ROS) [20]. It is likely that lysosomal storage causes immune activation by different molecular mechanisms, with an elevation of proinflammatory cytokines. Despite the fact that inflammation is a downstream event in the pathogenic cascade, it may nevertheless be a target for adjunctive therapy in multiple LSD. We, therefore, hypothesize the use of anti-inflammatory drugs, such as immunomodulators used in other LSD [22], as first- or second-line therapy. Because our patient developed secondary HLH, first-line treatment consisted of steroid drugs.

This speculation is consistent with the in-vitro immunomodulator results reported by Pavlova [9]. Dysregulation of autophagy can activate the inflammasome [20] and requires further investigation of whether it might be linked to HLH.

## 4. Conclusions

MPS-PS is a rare condition recently described, characterized by the absence of clear biological markers. MPS-PS should be considered in patients with the normal activity of lysosomal enzymes but with increased excretion of conjugated sialic acid, positive urinary sulfatides, and high plasma levels of heparan sulfate. The prognosis is generally unfavorable, and most of the patients reported in the literature have died before the age of 1–2 years. Currently, there is no specific therapy for MPS-PS, and its clinical management is limited to supportive and symptomatic treatment. Our patient presented with severe secondary HLH and was successfully treated with steroids. To date, this is the oldest patient affected by MPS-PS ever reported and the first one presenting with a secondary HLH. A favorable clinical course of the MPS-PS was observed in our patient, for whom undergoing a very prolonged and low-intensity steroid treatment apparently allowed a stabilization of his clinical conditions and an improved quality of life for him and his family. The favorable impact of very prolonged steroid treatment on the clinical course and the use of second-line immunomodulatory drugs deserves further investigation. We need to understand better the pathophysiology of this particular disorder that causes a dramatically rapid progressive multisystemic involvement.

## Figures and Tables

**Table 1:** Progressive tapering of steroid therapy: DXM—dexamethasone; HC—hydrocortisone.

Month	Steroid	Dosage	Dosage/Square Metre Per Day
Month 1	DXM	1.4 + 1.4 mg	6 mg/sqm
Month 3	DXM	1.3 + 1.3 mg	5 mg/sqm
Month 5	DXM	1.2 + 1.2 mg	4.7 mg/sqm
Month 6	DXM	1.1 + 1.1 mg	4.1 mg/sqm
Month 6	DXM	1 + 1 mg	3.7 mg/sqm
Month 8	DXM	0.9 + 0.9 mg	3.2 mg/sqm
Month 9	DXM	0.8 + 0.8 mg	2.8 mg/sqm
Month 10	DXM	0.5 + 0.5 mg	1.8 mg/sqm
Month 11	HC	10 + 10 + 10 mg	53.6 mg/sqm
Month 12	HC	10 + 10 + 5 mg	44 mg/sqm
Month 13	HC	10 + 10 mg	35 mg/sqm
Month 20	HC	7.5 + 7.5 mg	26.8 mg/sqm
Month 23	HC	5 + 5 mg	19 mg/sqm
Month 30 Ongoing	HC	5 + 2.5 mg	12.5 mg/sqm
Month 42	Synachten test		
